# Out-of-pocket payment and patients’ treatment choice for assisted reproductive technology by household income: a conjoint analysis using an online social research panel in Japan

**DOI:** 10.1186/s12913-022-08474-5

**Published:** 2022-08-27

**Authors:** Eri Maeda, Seung Chik Jwa, Yukiyo Kumazawa, Kazuki Saito, Arisa Iba, Ayako Yanagisawa‑Sugita, Akira Kuwahara, Hidekazu Saito, Yukihiro Terada, Takashi Fukuda, Osamu Ishihara, Yasuki Kobayashi

**Affiliations:** 1 Department of Environmental Health Science and Public Health, Akita University Graduate School of Medicine, 1-1-1 Hondo, Akita-shi, Akita, 010-8543 Japan; 2 Department of Obstetrics and Gynecology, Saitama Medical University, 38 Morohongo, Moroyama-machi, Iruma-gun, Saitama, 350-0495 Japan; 3 Department of Obstetrics and Gynecology, Akita University Graduate School of Medicine, 1-1-1 Hondo, Akita-shi, Akita, 010-8543 Japan; 4 Department of Comprehensive Reproductive Medicine, Graduate School, Tokyo Medical and Dental University, 1-5-45 Yushima, Bunkyo-ku, Tokyo, 113-8510 Japan; 5 Department of Public Health, Graduate School of Medicine, the University of Tokyo, 7‐3‐1 Hongo, Bunkyo‐ku, Tokyo, 113-0033 Japan; 6 Department of Obstetrics and Gynecology, Graduate School of Biomedical Sciences, Tokushima University, 3-18-15 Kuramoto-cho, Tokushima-shi, Tokushima, 770-8503 Japan; 7 Umegaoka Women’s Clinic, 1-33-3 Umegaoka, Setagaya-ku, Tokyo, 154-0022 Japan; 8 Center for Outcomes Research and Economic Evaluation for Health, National Institute of Public Health, 2-3-6 Minami, Wako-shi, Saitama, 351-0197 Japan

**Keywords:** Conjoint analyses, Public funding, Cost, Assisted reproductive technology, Socioeconomic factors

## Abstract

**Background:**

Economic disparities affect access to assisted reproductive technology (ART) treatment in many countries. At the time of this survey, Japan provided partial reimbursement for ART treatment only for those in low- or middle-income classes due to limited governmental budgets. However, the optimal level of financial support by income class remains unclear.

**Methods:**

We conducted a conjoint analysis of ART in Japan in January 2020. We recruited 824 women with fertility problems aged 25 to 44 years via an online social research panel. They completed a questionnaire of 16 hypothetical scenarios measuring six relevant ART attributes (i.e., out-of-pocket payment, pregnancy rate, risk of adverse effects, number of visits to outpatient clinics, consultation hours and kindness of staff) and their relations to treatment choice.

**Results:**

Mixed-effect logistic regression models showed that all six attributes significantly influenced treatment preferences, with participants valuing out-of-pocket payment the most, followed by pregnancy rates and kindness of staff. Significant interactions occurred between high household income (≥ 8 million JPY) and high out-of-pocket payment (≥ 500,000 JPY). However, the average marginal probability of the highest-income patients (i.e., ≥ 10 million JPY, ineligible for the subsidy) receiving ART treatment at the average cost of 400,000 JPY was 47%, compared to 56 − 61% of other income participants, who opted to receive ART at an average cost of 100,000 JPY after a 300,000 JPY subsidy.

**Conclusion:**

Our results suggest that out-of-pocket payment is the primary determinant in patients’ decision to opt for ART treatment. High-income patients were more likely to choose treatment, even at a high cost, but their income-based ineligibility for government financial support might discourage some from receiving treatment.

## Introduction

As more people postpone parenthood [1, 2], the use of assisted reproductive technology (ART) is increasing worldwide [3]. Despite the growing need and effectiveness of this treatment, economic factors have contributed to huge disparities in access to ART within and between countries [4–6]. To improve financial accessibility to ART treatments, governments of many countries provide various types of public funding, such as health insurance coverage, subsidies and tax refunds [7]. The proportions of reimbursement (i.e. full or partial) and eligibility criteria (e.g. clinical or demographical) vary substantially depending on regional and financial factors [7].

Japan provides universal health insurance coverage to the entire population [8]. In response to prolonged low fertility rates in Japan, ART treatments have been covered since April 2022 [9]. Prior to that date, only eligible couples received partial subsidies for ART treatment [10, 11]. Specifically, at the time of this survey in 2020, ART subsidies were available only to low- or middle-income patients. For high-income couples, the out-of-pocket payment for ART was approximately 400,000 − 500,000 JPY per cycle, or 3,300 − 4,200 euros using the 2020 exchange rate of 1 euro = 120 JPY [10, 11].

In Japan, eligibility rules for this and other subsidies (e.g. child allowance) often prioritize lower-income citizens [12]. Similar criteria were set up for ART subsidies in other East Asian countries, such as Korea [13] and Taiwan [14]. Yet, ART treatment is generally highly used among older and higher-income couples [15]. If these couples delay fertility treatment until they can better afford the associated cost, then the lack of subsidization for high-income couples could contribute to decreased rates of pregnancy [16]. In consideration of such potential adverse effects, the rationale for the eligibility criteria based on annual household income should be discussed.

Previous research has assessed the relationship between price and demand for ART [17], but few studies have assessed the quantitative association between demand and uptake of ART treatment and out-of-pocket payments by income level [4, 5]. The optimal amount of financial support by income class is unknown. We thus aimed to evaluate the probability of patients receiving ART treatment based on out-of-pocket payment and income class, using a conjoint analysis (CA).

## Methods

### Study setting

At the time of this survey in Japan, January 2020, the average cost per cycle of ART treatment was 380,000 JPY (i.e. 3,170 euros) for a fresh cycle and 510,000 JPY (i.e. 4,250 euros) for a freeze-all cycle, and these averages varied greatly across regions and institutions [18]. In lieu of health insurance coverage for ART treatment, the government offered partial reimbursement in the form of subsidies. The governmental subsidy was 300,000 JPY (i.e. 2,500 euros) for the first ART treatment cycle and 150,000 JPY (i.e. 1,250 euros) per cycle for the second to sixth cycles for legally married women younger than 40 (women aged 40–42 were limited to three cycles). Eligibility was income-based: annual household income after deducting predefined items could not exceed 7,300,000 JPY, corresponding to a gross income of 9,530,000 JPY (i.e. 79,400 euros).

### Participants and procedures

Participants were recruited via an online social research panel from the market research company Macromill (Tokyo, Japan), which manages a nationwide panel of more than 1 million registrants. To be considered for inclusion, participants had to be female, married, aged 25–44 years, currently experiencing infertility, and have a history of fertility tests or treatment. Macromill sent a pre-screening questionnaire to 210,212 randomly selected female registrants aged 25–44 years, then accepted pre-screening responses until reaching 20,000 respondents. Of those 20,000 respondents, 1,346 met the previously mentioned eligibility criteria. Macromill then sent recruitment emails to 1,247 who were randomly selected from the 1,346 eligible participants. Finally, 824 completed the survey and received a coupon (usually worth less than 1 Euro), consistent with Macromill’s procedures. All procedures were conducted from January 16 to 21, 2020. Participant responses were anonymous.

### Measures

#### CA questionnaire

**Fig. 1:**
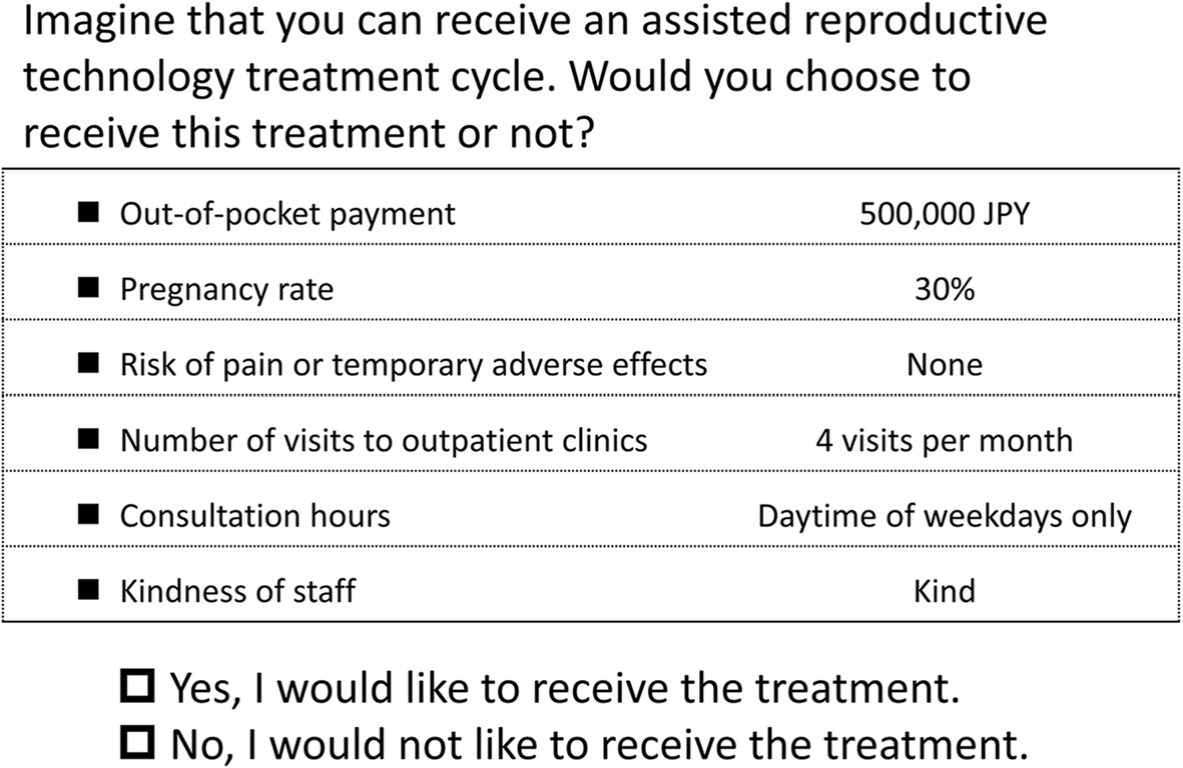
Example of a hypothetical scenario. JPY = Japanese Yen

Participants were presented with brief material explaining ART treatments, such as procedures, average pregnancy rates, possible adverse events due to medication and procedures, costs and subsidies and visits to outpatient clinics. We then presented hypothetical and realistic scenarios of ART treatment (see Fig. 1 for an example). As described in Table 1, scenarios comprised six relevant ART attributes (out-of-pocket payment, pregnancy rate, risk of adverse effects, number of monthly visits to outpatient clinics, consultation hours and kindness of staff). We selected these attributes based on literature about patient preferences in fertility care [19, 20], previous surveys conducted by a patient group in Japan [21], interviews with colleagues who experienced fertility care and a discussion with fertility specialists (SCJ, YKumazawa and OI) and public health researchers (EM, YKobayashi and TF). We divided each attribute into two to four levels covering a realistic range of Japanese fertility care settings. We established a range of out-of-pocket payments from 0 JPY (i.e. fully subsidised) to 800,000 JPY, or 6,700 euros (i.e. ineligible for subsidy). We used an orthogonal fractional factorial design to generate combinations of attribute levels, yielding 16 scenarios. Patients were asked to make ART treatment decisions in each of the 16 scenarios (see Fig. 1 for an example), which were presented in random order. To ensure that the questionnaire was understandable, we conducted a pilot survey on a small group of our colleagues, including those who experienced fertility problems.

**Table 1:** Attributes and corresponding levels

Attributes	Levels
Out-of-pocket payment	Free
	200,000 JPY
	500,000 JPY
	800,000 JPY
Pregnancy rate	5%
	15%
	30%
Risk of adverse effects	None
	Possible
Number of monthly clinic visits	4 visits
	8 visits
	12 visits
Consultation hours	Daytime of weekdays only
	Available at night and on weekends
Kindness of staff	Kind
	Not kind

*JPY* Japanese Yen

#### Socioeconomic and fertility status

Participant were grouped by age (25–29, 30–34, 35–39, or 40–44 years). They reported their annual household incomes (before deductions), educational backgrounds (university education, yes/no), average paid working hours per week (none, < 40 h, ≥ 40 h) and whether they had ever given birth (yes/no). We categorised annual household income into five groups (Low, < 4 million JPY; Lower-middle, 4–6 million JPY; Middle, 6–8 million JPY; Upper-middle, 8–10 million JPY; or High, ≥ 10 million JPY). The High-income group was assumed to be ineligible for governmental subsidy of ART treatments because only those with a gross household income of approximately 9.5 million JPY or lower were eligible. Participants reported their fertility information, including history (i.e., tests, timing methods, ovulation induction, intrauterine insemination, ART) and primary diagnosis (i.e., female factor, male factor, male and female factor, unknown, under investigation).

### Statistical analyses

Characteristics of the study participants were described and compared by household income class using Cuzick’s tests for trend. Using mixed-effects logistic regression models, we assessed 13,184 choices among 824 participants for the 16 ART treatment scenarios. Explanatory variables included the six previously described attributes of ART treatment (Model 1) and socioeconomic and fertility status (Model 2). In addition, we added to Model 2 an interaction term between household income and “out-of-pocket payment” and an interaction term between average working hours per week and “consultation hours” (Model 3).

To visualise all attribute effects, in Model 1, we calculated part-worth utility estimates using effect code (zero-centred) dummy variables, taking the value of -1 for the reference level [22]. To graph the relationships between treatment choice, out-of-pocket payment and income, we calculated in Model 1 the average marginal predicted probabilities by household income when the attribute “out-of-pocket payment” changed continuously. A two-sided *p-*value of < 0.05 indicated statistical significance. All analyses were performed using STATA14-MP (StataCorp LP, College Station, TX, USA) and R version 3.6.2 (R Foundation for Statistical Computing, Vienna, Austria).

### Ethical approval

The ethics committee at Akita University Graduate School of Medicine approved the study protocol (no. 2343) on December 20, 2019.

## Results

### Socioeconomic and clinical status of participants

Table 2 shows the background characteristics of the 824 female participants. Overall, most were in their thirties with middle-level household incomes, and 46% had a university education. About 36% had a child, and about 60% had paid work. Regarding fertility status, 31% had received ART treatments, but most (68%) were in early treatment stages (e.g. fertility tests, intrauterine insemination). The most frequent diagnosis of infertility was unknown (45%), followed by female factor (28%) and male and female factor (12%).

**Table 2:** Characteristics of the 824 participants (N, %) by household income class

	Total		Household income class ^a^										*P* for trend
			Low (*n* = 139)		Lower-middle (*n* = 266)		Middle (*n* = 251)		Upper-middle (*n* = 89)		High (*n* = 79)		
**Demographics and socioeconomic status**													
Age group													
25–29	100	(12.1)	18	(12.9)	35	(13.2)	31	(12.4)	12	(13.5)	4	(5.1)	0.39
30–34	294	(35.7)	41	(29.5)	108	(40.6)	88	(35.1)	28	(31.5)	29	(36.7)	
35–39	261	(31.7)	44	(31.7)	79	(29.7)	82	(32.7)	26	(29.2)	30	(38.0)	
40–44	169	(20.5)	36	(25.9)	44	(16.5)	50	(19.9)	23	(25.8)	16	(20.3)	
University education	379	(46.0)	42	(30.2)	92	(34.6)	139	(55.4)	50	(56.2)	56	(70.9)	< 0.001
Have a child	298	(36.2)	59	(42.4)	89	(33.5)	92	(36.7)	31	(34.8)	27	(34.2)	0.37
Paid work status													
None	329	(39.9)	84	(60.4)	117	(44.0)	91	(36.3)	24	(27.0)	13	(16.5)	< 0.001
< 40 working hours per week	307	(37.3)	43	(30.9)	120	(45.1)	92	(36.7)	29	(32.6)	23	(29.1)	
≥ 40 working hours per week	188	(22.8)	12	(8.6)	29	(10.9)	68	(27.1)	36	(40.4)	43	(54.4)	
**Fertility status**													
Treatment stage ^b^													
Receiving infertility tests with or without timed intercourse	346	(42.0)	64	(46.0)	115	(43.6)	90	(36.0)	42	(47.2)	35	(44.9)	0.43
Ovulation induction or intrauterine insemination	215	(26.1)	33	(23.7)	79	(29.9)	65	(26.0)	21	(23.6)	17	(21.8)	
Assisted reproductive technology treatments	259	(31.4)	42	(30.2)	70	(26.5)	95	(38.0)	26	(29.2)	26	(33.3)	
Infertility diagnosis													
Female	228	(27.7)	40	(28.8)	75	(28.2)	76	(30.3)	20	(22.5)	17	(21.5)	0.37
Male	65	(7.9)	11	(7.9)	13	(4.9)	22	(8.8)	12	(13.5)	7	(8.9)	
Male & female	100	(12.1)	13	(9.4)	38	(14.3)	35	(13.9)	6	(6.7)	8	(10.1)	
Unknown	374	(45.4)	68	(48.9)	118	(44.4)	104	(41.4)	44	(49.4)	40	(50.6)	
Under investigation	57	(6.9)	7	(5.0)	22	(8.3)	14	(5.6)	7	(7.9)	7	(8.9)	

*JPY* Japanese Yen

^a^ Low, < 4 million JPY, Lower-middle, 4–6 million JPY, Middle, 6–8 million JPY, Upper-middle, 8–10 million JPY, High, ≥ 10 million JPY

^b^ Four had missing data

The proportions of those having a university education and paid work were higher among higher income classes (*P*s for trend < 0.001). The other demographic and fertility status traits were not significantly different by income class, except for the small number of participants aged 25–29 in the High-income group.

### Relationships between attributes of treatment and choice of treatment

**Fig. 2:**
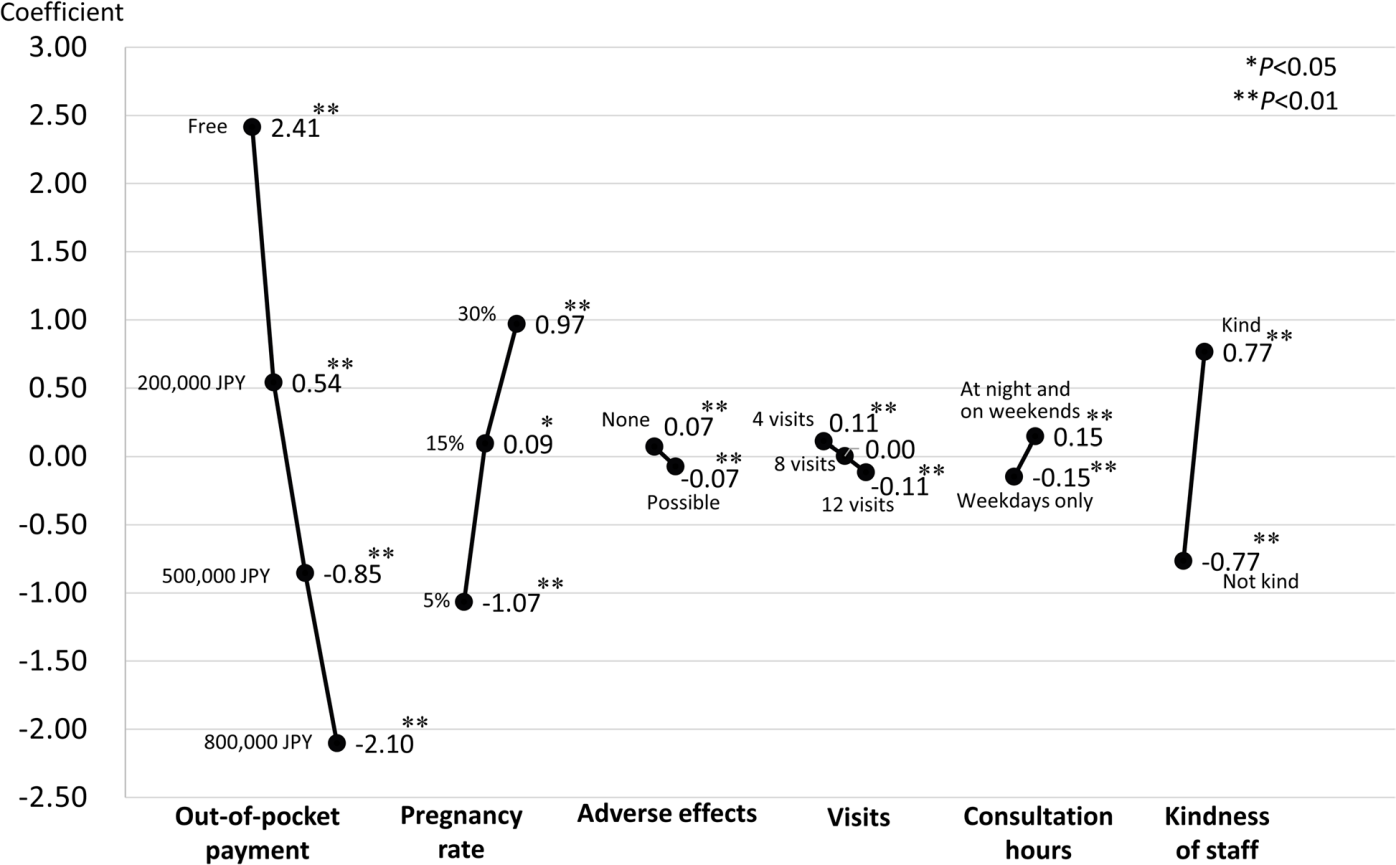
Part-worth estimates for levels of attributes of treatment. *P < 0.05, **P < 0.01. JPY = Japanese Yen

The mixed-effects logistic regression model showed that all six attributes were significantly associated with the choice to receive ART treatment (Model 1, Table 3 and Fig. 2). Participants were less likely to choose ART treatment when the out-of-pocket payment was high, when adverse events might happen, or when the monthly number of visits to the clinic was 12 (versus four) times. They were more likely to choose ART treatment when its associated pregnancy rates were high, when clinics were available at night and on weekends, or when staff were perceived as kind. Part-worth utility estimates suggested that participants valued out-of-pocket payment the most, followed by treatment pregnancy rates and kindness of staff (Fig. 2). The results were similar after adjusting for socioeconomic and clinical statuses of the participants (Model 2, Table 3). Those who had High household income, who worked 40 h per week, or who received more advanced treatments were more likely to choose ART treatments.

**Table 3:** Mixed-effects logistic regression analyses for factors related to the choice of receiving the assisted reproductive technology treatment (Models 1 and 2)

	Model 1				Model 2			
	Odds ratio	95% confidence intervals			Odds ratio	95% confidence intervals		
**Attributes of treatment**								
Out-of-pocket payment								
Free	Reference				Reference			
200,000 JPY	**0.154**	**0.133**	**―**	**0.178**	**0.153**	**0.132**	**―**	**0.177**
500,000 JPY	**0.038**	**0.032**	**―**	**0.045**	**0.038**	**0.032**	**―**	**0.045**
800,000 JPY	**0.011**	**0.009**	**―**	**0.013**	**0.011**	**0.009**	**―**	**0.013**
Pregnancy rate								
5%	Reference				Reference			
15%	**3.194**	**2.793**	**―**	**3.651**	**3.193**	**2.792**	**―**	**3.652**
30%	**7.676**	**6.656**	**―**	**8.854**	**7.657**	**6.637**	**―**	**8.834**
Risk of adverse effects								
None	Reference				Reference			
Possible	**0.865**	**0.778**	**―**	**0.961**	**0.865**	**0.779**	**―**	**0.962**
The number of visits to outpatient clinic								
4 times per month	Reference				Reference			
8 times per month	0.898	0.785	**―**	1.027	0.896	0.783	**―**	1.026
12 times per month	**0.797**	**0.688**	**―**	**0.925**	**0.797**	**0.687**	**―**	**0.924**
Consultation hours								
Daytime of weekdays only	Reference				Reference			
Available at night and on weekends	**1.343**	**1.209**	**―**	**1.492**	**1.347**	**1.212**	**―**	**1.497**
Kindness of staff								
Not kind	Reference				Reference			
Kind	**4.622**	**4.120**	**―**	**5.185**	**4.603**	**4.102**	**―**	**5.164**
**Socioeconomic status**								
Age group								
25–29					Reference			
30–34					1.139	0.696	**―**	1.863
35–39					0.655	0.395	**―**	1.084
40–44					1.253	0.724	**―**	2.169
University education								
No					Reference			
Yes					1.149	0.847	**―**	1.560
Household income class								
Low; < 4 million JPY					Reference			
Lower-middle; 4–6 million JPY					1.237	0.797	**―**	1.921
Middle; 6–8 million JPY					1.160	0.738	**―**	1.825
Upper-middle; 8–10 million JPY					1.587	0.887	**―**	2.840
High; ≥ 10 million JPY					**2.425**	**1.298**	**―**	**4.532**
Having a child								
No					Reference			
Yes					1.244	0.915	**―**	1.691
Paid work status								
None					Reference			
< 40 working hours per week					0.885	0.630	**―**	1.243
≥ 40 working hours per week					**1.663**	**1.094**	**―**	**2.528**
**Fertility status**								
Treatment stage								
Receiving infertility tests with or without timed intercourse					Reference			
Ovulation induction or intrauterine insemination					**1.451**	**1.004**	**―**	**2.098**
Assisted reproductive technology treatments					**4.537**	**3.158**	**―**	**6.518**
Infertility diagnosis								
Female					Reference			
Male					1.440	0.802	―	2.586
Male & female					1.009	0.615	―	1.654
Unknown					0.825	0.582	―	1.170
Under investigation					1.380	0.726	―	2.624

*JPY* Japanese Yen

The outcome is the choice of receiving the assisted reproductive technology treatment. Model 1 includes six attributes of assisted reproductive technology treatments as explanatory variables. Model 2 includes the six attributes and socioeconomic and fertility status

In Model 3, we observed significant interactions between the Upper-middle income class and an out-of-pocket payment of 500,000 JPY (*P* for interaction = 0.042) and between the High-income class and out-of-pocket payments of 500,000 JPY or 800,000 JPY (both *Ps* for interaction < 0.001), as shown in Table 4. We also observed a significant interaction between working more than 40 h per week and clinic consultation availability on nights and weekends (*P* for interaction = 0.005).

**Table 4:** Mixed-effects logistic regression analyses for factors related to the choice of receiving the assisted reproductive technology treatment. Results from Model 3 include interaction terms between household income and "out-of-pocket payment" and between paid work status and “consultation hours”

	**Odds ratios of “out-of-pocket payment”**				
Household income class	Free	200,000 JPY	500,000 JPY		800,000 JPY
Low; < 4 million JPY	Reference	0.136 (0.096 − 0.192)	0.025 (0.017 − 0.037)		0.008 (0.005 − 0.013)
Lower-middle; 4–6 million JPY	Reference	0.142 (0.110 − 0.182)	0.028 (0.021 − 0.038)		0.007 (0.005 − 0.010)
Middle; 6–8 million JPY	Reference	0.164 (0.128 − 0.211)	0.039 (0.030 − 0.052)		0.010 (0.007 − 0.014)
Upper-middle; 8–10 million JPY	Reference	0.129 (0.085 − 0.197)	**0.046 (0.029** − 0.072)^*^		0.015 (0.009 − 0.025)
High; ≥ 10 million JPY	Reference	0.221 (0.142 − 0.343)	**0.109 (0.068** − 0.174)^**^		**0.038 (0.023** − 0.062)^**^
	**Odds ratios of “consultation hours”**				
Paid work status	Daytime of weekdays only			Available at night and on weekends	
No paid work	Reference			1.187 (1.006 − 1.399)	
< 40 h per week	Reference			1.306 (1.100 − 1.551)	
≥ 40 h per week	Reference			**1.736 (1.403** − 2.148)^**^	

The outcome is the choice of receiving the assisted reproductive technology treatment. Model 3 includes six attributes of assisted reproductive technology treatments, socioeconomic and fertility status of the participants, an interaction term between household income and "out-of-pocket payment" and an interaction term between paid work status and “consultation hours”

^*^ P for interaction < 0.05. ^**^P for interaction < 0.01. JPY = Japanese Yen

**Fig. 3:**
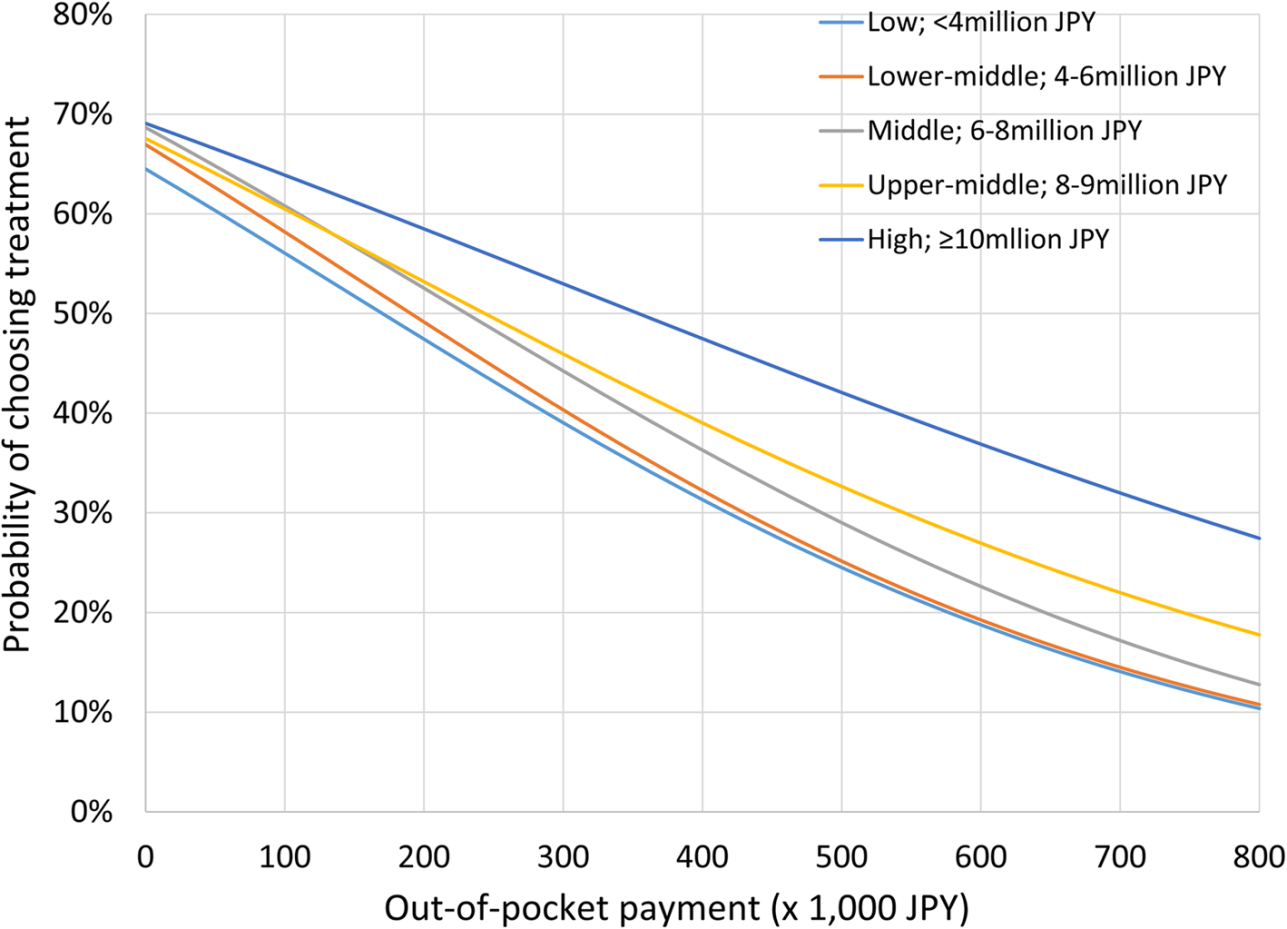
Probability of choosing assisted reproductive technology treatment by income class when out-of-pocket payment changes. JPY = Japanese Yen

Figure 3 shows the average marginal probabilities of opting for ART treatment across the income groups in Model 1, when the out-of-pocket payment changed continuously. The probability of opting for ART was consistently higher among the High-income group, compared to other income groups. For example, for the scenario in which an initial ART cycle cost 400,000 JPY, the average marginal probability of High-income participants choosing treatment was 47%. In the same scenario but including an income-based subsidy of 300,000 JPY (reducing the out-of-pocket cost to 100,000 JPY) the average marginal probability of opting for ART treatment was 56% among Low-income participants and 61% for Upper-middle-income participants. These probabilities decreased to 43% for Low-income participants and 50% for Upper-middle-income participants for the second through sixth treatments, for which subsidies decreased to 150,000 JPY (i.e. 250,000 JPY out-of-pocket) each.

## Discussion

In our CA study, we found that the out-of-pocket payment was the most influential determinant of ART treatment choice among participants, though all six attributes (e.g. pregnancy rates and kindness of staff) significantly influenced treatment preference. Higher-income patients were more likely to receive ART treatment even at a high cost, but their ineligibility for financial support due to their high income might discourage some from receiving treatment. This quantitative evaluation suggested that patients’ willingness to receive ART treatment could change substantially according to public funding of ART treatment. Many countries have sought to improve treatment accessibility in the context of limited financial resources, leading to enormous fluctuations in the number of treatments that patients opt to receive [4, 23, 24]. CA could be a feasible way to help policymakers identify the most appropriate programs for public funding under fiscal constraints.

Among the various factors affecting fertility care utilisation (e.g. sociocultural acceptability, availability), financial accessibility is a dominant one [6, 11]. Our findings corroborate that patients consider their out-of-pocket payment to be the most important attribute in their fertility decisions (Fig. 2). In addition, our prediction is consistent with previous findings on the relationship between cost and utilisation of ART treatment. For example, in an ecological study of 30 high-　and　upper-middle-income countries, Chambers et al. [5] find that a 1 percentage point decrease (based on annual disposable income for a single person with no dependents) in the cost of a treatment cycle predicts a 3.2% increase in utilisation. In our study, the probability of participants choosing ART treatment increased by 54% when the out-of-pocket payment decreased by 800,000 JPY (Fig. 3), corresponding with a 2.3% increase in demand per 1 percentage point decrease (based on disposable household income) in cost [25]. However, the slope differed depending on both income and out-of-pocket payment.

A study on ART treatment copayments in Germany demonstrated arc price elasticity of demand of -0.36 [17]: in other words, when the copayment for fertility treatment increased from free to 50% (1,500–2,000 euros), ART treatments dropped by 53% [23]. We observe a smaller elasticity (-0.13) but comparable inelasticity when the cost of ART treatment increases from 0 (free) to 200,000 JPY (1,670 euros), which is similar to results for other medical services, generally in the range of -0.1 to -0.3 [26]. Although the choices made in a CA would not completely match actual treatment choices, we confirmed that the CA could reasonably predict treatment choices based on arbitrary treatment costs.

As shown in Fig. 3, higher-income participants consistently opted for ART even at a higher cost: 27% of those in the High-income group were willing to pay up to 800,000 JPY (6,770 euros). For out-of-pocket payments exceeding 500,000 JPY (4,170 euros), we observed significant interactions between out-of-pocket payments and the Upper-middle- or High-household income groups (Table 4), suggesting an important effect of income: The negative impact of cost on ART demand was lower among higher-income populations, compared to lower-income populations.

Yet, the probability of receiving ART treatment among High-income group could fall below that of other income groups, depending on their subsidy eligibility. Among all five income groups, to attain a 47% probability of opting for ART treatment costing 400,000 JPY (as we observed for the High-income group), the ideal subsidy amounts are 200,000 JPY for Low-, 180,000 JPY for Lower-middle, 140,000 JPY for Middle- and 120,000 JPY for Upper-middle income groups. In 2020, the Japanese government provided a 150,000 JPY income-based subsidy for each of the second through sixth ART treatments, which was close to our suggested ideal amount. In contrast, the 300,000 JPY subsidy for the first application may induce a sense of “unfairness” among High-income patients.

In response to the country’s prolonged low fertility rate, the Japanese government removed the income eligibility criteria at the end of 2020 [10]. Since April 2022, up to six (three) cycles of ART treatments have been covered by health insurance for all legally or virtually married women aged < 40 years (40–42 years). Under the health insurance scheme, out-of-pocket payment for a fresh ART treatment cycle is estimated to be approximately 150,000 JPY (e.g., when six to nine eggs are retrieved, and two to five embryos are cultured and cryopreserved) [9]. This policy change helps to eliminate the perceived “unfairness” among High-income couples and to increase the total number of fertility treatments that women receive. Our model calculated that the probability of High-income couples opting for a fresh ART treatment cycle would increase nearly 1.3 times, from 47 to 61%, if the out-of-pocket payment decreased from 400,000 JPY(i.e., without a subsidy) to 150,000 JPY(i.e., covered by health insurance). This potentially large fiscal impact should be monitored to ensure that public funding for ART remains sustainable.

Interestingly, we found that Japanese patients preferred to seek ART treatment when the clinic staff was perceived as friendly (Fig. 2). Our findings agree with studies in China [19] and Europe [27] indicating that patients value physician’s attitude toward patients as much as they value the treatment’s success rate, whereas physicians underestimate the importance of patient-centred care. Although we could not assess a variety of aspects of patient-centredness, such as physician continuity or information provision of treatment [28], this is the first study to quantify the effects of patient-centred fertility care on treatment choice of patients in Japan. The fact that patients attached great importance to staff attitudes should promote clinicians’ understanding of the care they seek.

Another important finding of this study relates to women engaged in paid work for 40 h or more per week, who were more likely to choose treatment if they could visit outpatient clinics at night or on weekends. Aligned with rises in parental age and women’s labour force participation [2], 60% of women receiving fertility care have concerns about missing work [29], and about 17% of women in Japan resign from their jobs after starting fertility treatment [30]. We found that medical institutions offering consultation during nights and weekends were preferred by participants working outside the home. Since ART procedures require frequent and sometimes unpredictable visits based on the menstrual cycle [30], providing flexible clinic hours and a supportive work environment would help employees balance their fertility treatments with their work schedules [31].

This study has some limitations. First, attributes not included in our CA scenarios might be relevant in real-life settings. We selected six attributes based on surveys in Japan [21] and abroad [19, 20] and on our interviews of patients and clinicians. To maintain a reasonable number of scenarios presented to participants, we did not include all possible attributes (e.g. geographical access to the institution, amount of time required to see doctors). However, we ensured through the pilot survey that the questionnaire was understandable and that no vital information was missing from the scenarios. A future qualitative study including in-depth interviews in Japan might help construct a CA questionnaire. Second, decisions in a CA may not exactly match those during actual treatment. Empirical studies on patients’ actual behaviours before and after the subsidy policy revision and based on health insurance coverage could help confirm the validity of this study. Third, the use of social research panels could have caused selection bias associated with higher education [32, 33]. To recruit eligible samples efficiently, the market research company invited a large sample to the pre-screening, accepted responses in order of arrival and invited randomly selected participants to the survey. However, the sociodemographic distribution of our participants was similar to that of previous clinical settings in Japan [30], and patients with fertility problems tend to have higher education levels. Thus, our data should not be heavily influenced by potential selection bias. Finally, this study focused on ART treatment in Japan. However, CA is a generic approach that can be used widely. Future research should assess cultural relevance in perceptions of fertility information.

## Conclusions

We found that out-of-pocket payments were the most influential determinant for patients in Japan seeking ART treatment. Higher-income patients opted for treatment even at a higher cost, but their income-based ineligibility for government financial support discouraged some from receiving treatment. Although the setting of the present study was in Japan, CA could be a feasible way to discuss appropriate programs for public funding in many countries.

## Data Availability

The datasets generated and/or analyzed during the current study are not publicly available due to participant privacy, but they are available from the corresponding author on reasonable request.

## Abbreviations

ART: Assisted reproductive technology
CA: Conjoint analysis
JPY: Japanese Yen
